# Advances in research on novel technologies for the detection of exogenous contaminants in traditional Chinese medicine

**DOI:** 10.3389/fphar.2025.1658241

**Published:** 2025-08-14

**Authors:** Ziyu Guo, Junyao Li, Lina Zeng, Ping Wang, Meifang Li, Chang Su, Shuhong Wang

**Affiliations:** Shenzhen Institute for Drug Control, Shenzhen, China

**Keywords:** Chinese materia medica, exogenous contaminants, novel technologies and methods, biosensor, quality of traditional Chinese medicine

## Abstract

Exogenous contaminants in traditional Chinese medicine (TCM), including pesticide residues, heavy metals, mycotoxins, and sulfur dioxide residues, pose significant risks to human health and environmental safety. Conventional detection methods are limited by insufficient sensitivity, complex sample preparation, and challenges in multi-residue analysis, compromising accuracy and efficiency. To address these critical bottlenecks—particularly the escalating regulatory demands and trade barriers due to contamination incidents—this review establishes the first integrated ‘dual track’ quality control framework for TCM contaminants. We propose a novel risk stratified strategy synergizing laboratory grade accuracy with field deployable screening, overcoming the sensitivity portability trade-off. This work provides a roadmap for establishing globally harmonized standards. Future research should prioritize high-throughput methods, intelligent analytics, and green detection technologies. Integrating AI-driven automation with data traceability could establish unified systems for contaminant detection and degradation, enhancing TCM quality control and global competitiveness.

## 1 Introduction

The rising global demand for Traditional Chinese Medicine (TCM) underscores the critical importance of its quality and safety. Contamination by exogenous contaminants—including pesticide residues, heavy metals, mycotoxins, and sulfur dioxide residues (Wang et al., 2015; Zuo et al., 2024) during cultivation, processing, storage, or transportation poses significant risks to TCM integrity. These contaminants not only compromise therapeutic efficacy but also threaten patient health through acute/chronic toxicity and immune dysfunction. Alarmingly, over 80% of 31 rejected Chinese herbal export batches in early 2020 were attributed to excessive pesticide and heavy metal levels (Sun et al., 2022), highlighting this issue as a major regulatory and societal concern. Furthermore, the globalization of TCM trade has intensified the challenge of regulatory frameworks: the European Union sets maximum level for certain contaminants with the Commission Regulation (EC) No 1881/2006, while FDA emphasizes toxicological risk assessment in Botanical Drug Guidance, and the Chinese Pharmacopoeia continues to update contaminant standards. Therefore, the imperative to address exogenous contaminants in TCM extends beyond immediate safety concerns to encompass global regulatory compliance, industrial sustainability, and international market access. Establishing internationally aligned quality control systems is therefore essential to ensure the safety of TCM and global market competitiveness. Within this framework, advancing detection technologies serve as the cornerstone for effective quality control, where accurate, rapid, and sensitive methods are crucial for mitigating contaminants.

However, the development and application of advanced detection technologies must overcome numerous challenges. The intrinsic chemical diversity and complexity of traditional Chinese medicine (TCM) matrices present significant obstacles to the efficient extraction, purification, and definitive detection of trace contaminants. Conventional analytical techniques often struggle under such conditions, encountering severe matrix effects in mass spectrometry, spectral interferences in spectroscopy, and non-specific binding in immunoassay procedures. Moreover, the profile of contaminants is continuously evolving, emerging pollutants such as perfluoroalkyl substances (Xing et al., 2023) and phthalates (Huang et al., 2021) introduce distinct analytical difficulties due to their ultra-trace concentrations and novel molecular structures. Importantly, increasingly stringent and evolving international regulatory requirements necessitate analytical platforms that are not only highly sensitive and specific but also robust, high-throughput, and capable of multi-residue analysis. The shortcomings of current technologies-including insufficient sensitivity for detecting ultra-trace novel toxins, limited throughput for large-scale monitoring, susceptibility to complex matrix effects, lack of portability for on-site applications, and high operational costs-collectively highlight the pressing need for innovative and transformative solutions.

In this review, we systematically summarized the principles and matrix adaptation strategies of these emerging technologies, not only fills the void in current literature that often focuses on single-method evaluations but also provides a roadmap for harmonizing detection protocols with global regulatory standards. This analysis aims to inform the development of next-generation detection strategies and support the establishment of risk early-warning systems for TCM contaminants.

## 2 Classification of exogenous contaminations in TCM

**FIGURE 1:**
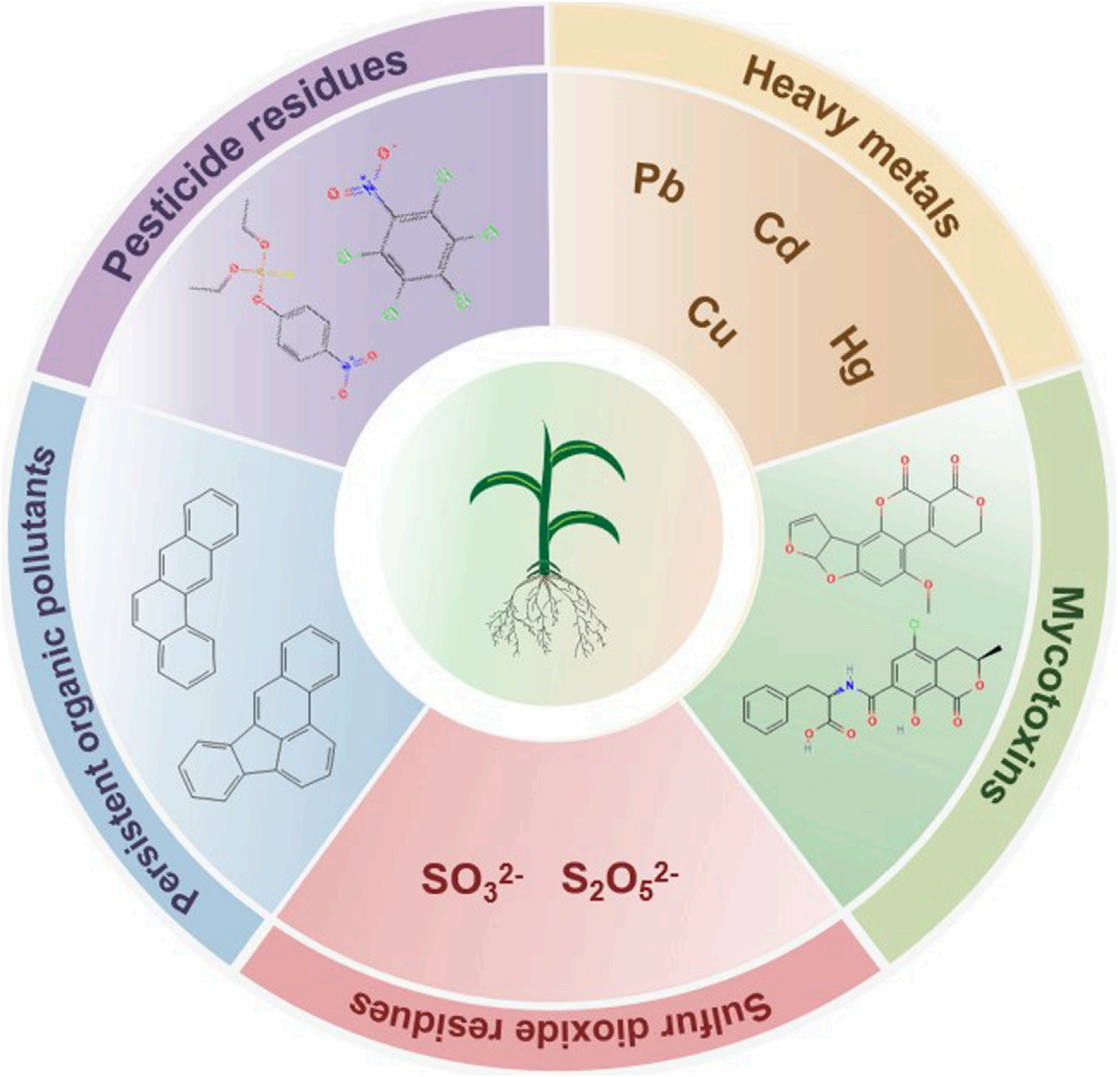
Schematic classification of exogenous contaminants in TCM.

Exogenous contaminations in TCM refer to harmful substances introduced from the external environment during the processes of growth, processing, storage, and transportation, which may adversely affect the quality, safety, and efficacy of TCM (Wang Y. et al., 2025). They primarily include pesticides residues, heavy metals, mycotoxins, sulfur dioxide residues, and other organic pollutants (Figure 1).

### 2.1 Pesticide residues

Pesticide residues encompass residual prototypes, metabolites, and degradation products remaining after pesticide application for pest control during TCM cultivation. Common classes include organochlorines, organophosphates, pyrethroids, carbamates, and neonicotinoids (Chen et al., 2022). Long-term exposure poses significant health risks due to cumulative toxicity and disruption of physiological functions (e.g., reproductive, endocrine, neurological, hepatic systems). Organochlorine and organophosphate pesticides exhibit the highest detection rates in TCM. Organochlorine contamination is prevalent in the perennial roots and rhizomes, while organophosphate pollution predominantly affects the aerial parts such as flowers, leaves, and whole plants (Li et al., 2019).

### 2.2 Heavy metals

Heavy metals, characterized by high density and toxicity, include lead (Pb), cadmium (Cd), mercury (Hg), and copper (Cu). Arsenic (As) is often classified alongside due to similar toxicity, sources, and health risks (Jomova et al., 2025). Residues in TCM primarily arise from environmental pollution (soil, water, air) and agricultural equipment. These elements are highly toxic, persistent, and can disrupt protein/enzyme structures, inhibiting their activity. They can damage the nervous, immune, and digestive systems, with long-term exposure potentially leading to carcinogenesis or death (Kim et al., 2019). Analysis of 1773 batches from 86 medicinal plants revealed 30.51% exceeded regulatory limits for at least one heavy metal, with exceedance rates ranked Pb > Cd > As > Hg > Cu (Luo et al., 2021).

### 2.3 Mycotoxins

Mycotoxins can be produced by fungi during the cultivation, harvesting, storage, or processing of Chinese materia medica (Zhao D. T. et al., 2021). Over 400 mycotoxins and metabolites are known to exist. Aflatoxins, ochratoxin A (OTA), zearalenone, deoxynivalenol, and fumonisins are of particular concern due to high toxicity and contamination rates (Al-Jaal et al., 2019), exhibiting significant carcinogenic, teratogenic, and mutagenic effects. Aflatoxin contamination is common in seeds, roots and rhizomes, animal-origin TCM and fermented materials. OTA shows high rates in *Glycyrrhiza uralensis* and *Myristica fragrans*, zearalenone in *Coicis Semen*, and fumonisins in *Panax notoginseng* and *Pheretima* (Zhou et al., 2022).

### 2.4 Sulfur dioxide residues

Sulfur fumigation, commonly employed for bleaching, preservation, and drying during TCM processing, can lead to elevated sulfur dioxide residues and degradation of active components. Absorbed sulfur dioxide forms sulfite derivatives, potentially damaging skin mucosa, respiratory, nervous, cardiovascular, and reproductive systems, and increasing risks of asthma, cardiovascular diseases, and chronic lung conditions (Wang T. et al., 2024). TCM materials with high starch and polysaccharide contents, such as *Angelicae Dahuricae* Radix, are frequently subjected to sulfur fumigation, often resulting in excessive sulfur dioxide residues (Kang et al., 2018).

### 2.5 Persistent organic pollutants

Persistent organic pollutants (POPs) in TCM are characterized by environmental persistence, resistance to degradation, and human health risks. They infiltrate plants though their roots from contaminated soil or via the aerial parts from the atmosphere, and can be introduced during processing. Common POPs include carcinogenic, teratogenic, and mutagenic polycyclic aromatic hydrocarbons (PAHs) and polychlorinated biphenyls (PCBs) (Wang L. et al., 2025).

## 3 Detection technologies for exogenous contaminants in TCM

The heterogeneity of exogenous contaminants in TCMs necessitates distinct analytical approaches dictated by their physicochemical properties (Table 1). Organics—including pesticide residues, mycotoxins, and polycyclic aromatic hydrocarbons (PAHs)—share similar extraction protocols and chromatographic behaviors, positioning liquid chromatography-tandem mass spectrometry (LC-MS/MS) as the gold standard for multi-residue analysis with unparalleled sensitivity and selectivity. Concurrently, biosensing platforms (e.g., aptasensors, immunoassay arrays) are emerging as rapid, cost-effective solutions for on-site screening applications. Heavy metals (Pb, Cd, As, etc.) require atomic spectroscopic techniques: inductively coupled plasma mass spectrometry (ICP-MS) enables ultra-trace multi-element quantification, while atomic absorption spectroscopy (AAS) provides precise speciation analysis. Sulfur dioxide residues demands specialized methodologies such as ion chromatography.

**TABLE 1:** Comparative analysis of detection technologies for exogenous contaminants in TCM.

Technology category	Specific technology	Target contaminants	Advantages	Limitations
Chromatography	Chromatography-mass spectrometry	Pesticide residues, Mycotoxins	High sensitivity, Multi-residue analysis, Structural confirmation capability	Expensive instrumentation cost, Complex sample pretreatment, Significant matrix effects, Requires specialized operators
	High-performance liquid chromatography-fluorescence detector (HPLC-FLD)	Mycotoxins, PAHs	Applied to compounds with fluorescent groups	Limited separation efficiency, Long analysis time
	Ion chromatography (IC)	Sulfites	Applied to ionic compounds, Low reagent consumption	Complex sample pretreatment, High instrumentation cost
Spectroscopy	Atomic absorption spectroscopy (AAS)	Heavy metals	Simple operation, Low cost, Accurate single-element quantification	Single-element detection, Complex pretreatment
	Atomic fluorescence spectroscopy (AFS)	As, Hg	High sensitivity, Strong anti-interference capability	Limited to specific elements, Fluorescence quenching, Narrow measurement range, High requirements for instrument stability
	Near-infrared spectroscopy (NIRS)	Pesticides, Mycotoxins	Nondestructive, Rapid, No pretreatment, Field-deployable	Low sensitivity, Model-dependent requiring large sample sets, Model overfitting
	Terahertz time-domain spectroscopy (THz-TDS)	Pesticides, Mycotoxins	Non-ionizing radiation, Fingerprint identification, Strong penetration	Strong water absorption interference, Expensive instrumentation cost
	Surface-enhanced Raman spectroscopy (SERS)	Pesticides, SO_2_, Mycotoxins, PAHs	High sensitivity, Fingerprint information, Wide applicability, Rapid detection	Poor substrate reproducibility, Significant substrate dependence, Expensive instrumentation cost
	Hyperspectral imaging (HSI)	Mycotoxins, Heavy metals, Pesticides	Image-spectrum fusion, Visual localization of contaminants	Complex data processing, High hardware costs
Mass Spectrometry	Inductively coupled plasma mass spectrometry (ICP-MS)	Multi-element heavy metals	Multi-element detection, Ultra-trace analysis	Expensive instrumentation, Complex pretreatment
Biological Detection	Enzyme inhibition	Organophosphates, Carbamates	Rapid, Low-cost, Simple operation	Limited to specific pesticides, Enzyme activity inhibition, High false-positive rate
	Enzyme-linked immunosorbent assay (ELISA)	Pesticides, Mycotoxins, PAHs	High specificity and sensitivity	Lengthy antibody development, Cross-reactivity interference
	Colloidal gold immunoassay		Rapid on-site detection, Instrument-free	Qualitative/semi-quantitative, Lower sensitivity than ELISA
	Fluorescent immunoassay		Enhanced sensitivity, Quantitative capability	Requires fluorometers, Higher cost, Matrix interference
	Immunochromatography (ICA)		Rapid on-site detection, Multi-channel detection	Cross-reactivity interference, Insufficient stability
	Molecularly imprinted polymers (MIP)	Pesticides, Mycotoxins, PAHs	High selectivity and specificity, Reusability	Limited adsorption capacity, High preparation requirements
	Aptamers	Mycotoxins, Sulfite derivatives	High selectivity and specificity, Flexible chemical modification	Complex screening, High environmental sensitivity
Biosensors	Nanomaterial-based biosensors	Mycotoxins, Pesticides	High selectivity and specificity, Real-time monitoring, Portability	Short lifespan, Matrix interference
	Microfluidic-based biosensors		Integration capability, Multi-channel detection	High cost, Technical complexity
	Dual-mode biosensors		Signal complementarity enhances reliability	Complex structure, Limited stability

Note: The “high sensitivity” mentioned in this table generally refers to the ppb level, while the “ultra-trace analysis” in the ICP column can reach 0.001 ppb.

### 3.1 Chromatography

Chromatography, a traditional detection method, plays an irreplaceable role in screening pesticide residues, mycotoxins, and organic pollutants due to its high separation efficiency, sensitivity, and broad applicability.

#### 3.1.1 Liquid chromatography

Mycotoxins with natural fluorescence, such as aflatoxin B1 (AFB1) and OTA, can be detected selectively using HPLC with fluorescence detection (HPLC-FLD). For mycotoxins lacking fluorescent groups, such as fumonisin B1 (FB1), derivatization is required to introduce chromogenic or fluorescent groups to the analyte for subsequent detection (Maneeboon et al., 2023). Fluorescent PAHs are also suitable for HPLC-FLD. Yu et al. (2022) developed an HPLC-FLD method using a C18 reversed-phase chromatographic column, achieving a detection limit of approximately 1 ng/mL for six PAHs, which was 10–100 times lower than that of UV detection. Ultrasound-assisted liquid-liquid extraction combined with HPLC-FLD was employed to analyze four PAHs (benzo [a]anthracene, benzo [b]fluoranthene, anthracene, and benzo [a]pyrene) in 70 TCM varieties; all samples contained at least one PAH, with highest BaP (4.82 μg/kg) and PAH4 (9.60 μg/kg) levels in Angelicae sinensis Radix (Kim and Shin, 2022). It is worth noting that pigmented extracts can cause fluorescence quenching, necessitating SPE or gel permeation chromatography (GPC) for purification. Benny et al. (2021) employed a modified SPE pretreatment method in conjunction with HPLC-FLD to quantitatively determine PAHs in aqueous-alcohol extracts of purslane and hedyotis. The SPE purification technique significantly reduced the number and types of co-extracted compounds, achieving recovery rates ranging from 92% to 98%.

#### 3.1.2 Ion chromatography

Ion chromatography (IC), as the predominant method for detecting sulfur dioxide residues in TCM, primarily relies on a conductivity detector to quantify ion concentrations by measuring changes in solution conductivity. A key advantage is its ability to directly determine sulfite ions (SO_3_^2-^) without requiring derivatization. Tang and Wang (2021) analyzed sulfur dioxide residues in foods such as mushrooms and tremella, achieving recovery rates from 69.7% to 109.9%, with detection limits of 0.10–1.00 mg kg^-1^. Yang et al. (2019) developed an accurate quantitative analysis method for sulfite determination in fresh meat and shrimp using IC coupled with inhibition conductivity detection, achieving LOD/LOQ of 2.7 mg/kg and 8.2 mg/kg, and recoveries of 85%–92%. Liu et al. (2020) employed ion chromatography to determine the sulfur dioxide residues content in angelica with the LOD reaching 0.02 mg/kg.

#### 3.1.3 Chromatography-mass spectrometry

Chromatography-mass spectrometry is widely regarded as the gold standard for pesticide residue detection, combining exceptional chromatographic separation with the specificity of mass spectrometry. Gas chromatography (GC) excels for volatile organochlorines (e.g., DDT, hexachlorocyclohexane) and pyrethroids, while liquid chromatography (LC) is suitable for thermally unstable or polar pesticides (e.g., methamidophos, glyphosate). Tandem mass spectrometry (LC-MS/MS, GC-MS/MS) enhances capabilities by providing structural identification and molecular mass information, overcoming limitations of HPLC for multi-residue screening without reference standards. Complex TCM matrices often require pretreatment (e.g., QuEChERS, solid-phase extraction (SPE)) to minimize interference (Basij et al., 2025; Park et al., 2018). With the rapid advancement of high-sensitivity and high-resolution mass spectrometry technologies, ultra-high performance liquid chromatography-triple quadrupole time-of-flight mass spectrometry (UHPLC-Q-TOF/MS) is increasingly being adopted for the rapid screening and analysis of unknown pesticide residues (Zhang W. et al., 2024). Nakagawa et al. (2011) utilized LC-Orbitrap MS, leveraging the precise measurement of characteristic ion masses and MS/MS fragmentation spectra, to identify a novel Fusarium mycotoxin glucoside. Mass spectrometry remains the most widely used detection method for pesticides and mycotoxins due to its high sensitivity and accuracy. However, the high cost and operational complexity of MS limit its universal application.

### 3.2 Spectroscopy

Spectroscopy offers high sensitivity, selectivity, and rapid nondestructive detection, and can accurately identify and quantitatively analyze pollutants such as pesticide residues, heavy metals, and mycotoxins, playing a key role in the detection of exogenous contaminants in TCM.

#### 3.2.1 Atomic absorption spectroscopy

Atomic absorption spectroscopy (AAS) determines the concentration of metal elements by exploiting the absorption characteristics of atomic vapor at specific wavelengths of light. Owing to its high sensitivity and low detection limits, AAS is widely used in the analysis of trace heavy metals. However, stringent requirements exist for sample pretreatment; for example, interfering elements within the sample matrix must be mitigated using background correction techniques. Furthermore, AAS is not suitable for the simultaneous determination of multiple elements. Microwave digestion pretreatment technology can substantially reduce reagent consumption and minimize matrix interference (Xu et al., 2019). Flame atomic absorption spectrometry (FAAS) is well-suited for the rapid analysis of high-concentration elements (e.g., Cu, Fe), whereas graphite furnace atomic absorption spectrometry (GFAAS), with μg/kg sensitivity via electrothermal atomization, is ideal for trace elements (e.g., Pb, Cd) (Wen et al., 2021). The single-element detection mode of AAS limits its high-throughput application, necessitating the use of hydride generation (HG) technology to enhance signals and ensure sensitivity (Wu et al., 2015).

#### 3.2.2 Atomic fluorescence spectroscopy

Atomic fluorescence spectroscopy (AFS) excites atoms or molecules in the sample and determines the concentration of elements by measuring the emitted fluorescence signals. It offers high sensitivity, particularly for hydride-forming elements like Hg and As. While enabling multi-metal detection, it is limited by saturation effects and fluorescence quantum efficiency (Wu X. et al., 2022). Cold vapor atomic fluorescence spectrometry (CV-AFS) uses stannous chloride (SnCl_2_) to reduce Hg^2+^ in solution to mercury vapor, which is subsequently introduced into a quartz atomizer for cold atomic fluorescence measurement. This method overcomes the disadvantages of HG-AFS for mercury determination, such as the thermal excitation-induced reduction of ground-state mercury atoms by the argon-hydrogen flame and noise caused by the flame (Chen et al., 2014). Wei et al. (2025) developed a hydrogen peroxide (H2O2)-assisted microwave digestion-sequential injection CV-AFS method for determining total Hg content in eight TCMs, including Dendrobii Caulis, achieving an LOD of 0.011 μg/L. It is important to note that AFS is only applicable to specific elements (e.g., Hg, As, Se), and the concentration of the reducing agent and the carrier gas flow rate must be strictly controlled to avoid fluorescence quenching.

#### 3.2.3 Near-infrared spectroscopy

Near-infrared spectroscopy (NIRS) is grounded in the theory of molecular vibrational energy level transitions. By collecting diffuse reflection or transmission spectral information from samples within the 780–2500 nm band and integrating it with chemometric models (e.g., principal component analysis), a quantitative correlation model can be established between spectral characteristics and pollutant concentrations, enabling the rapid screening of exogenous pollutants in TCM. NIRS eliminates the need for complex sample pretreatment and avoids the consumption of organic solvents, aligning with the principles of green analytical chemistry. Furthermore, its nondestructive detection process preserves the integrity of medicinal materials, making it particularly suitable for online real-time monitoring applications. Zhang et al. (2023b) used NIRS (940–1660 nm) for AFB1 in peanuts, achieving >91% accuracy and AUC>0.90 after optimization. Nazarloo et al. (2021) employed Vis/NIRS with Partial Least Squares Regression (PLSR) and Artificial Neural Networks (ANN) models for dynamic, nondestructive pesticide detection in tomatoes. Zhang et al. (2023a) used Vis/NIRS with Partial Least Squares Discriminant Analysis (PLS-DA) and Least Squares Support Vector Machine (LS-SVM) to detect abamectin, dichlorvos, and carbendazim on cauliflower, achieving 98.33% accuracy. The limitations of NIRS include: model establishment depends on extensive, representative sample libraries and requires regular updates to address matrix variations; sensitivity to trace pollutants is inferior compared to mass spectrometry. Complex spectral analysis necessitates professional chemometrics support and carries the risk of model overfitting. Current research focuses on enhancing sensitivity via nano-enhanced substrates or deep learning algorithms (Lapcharoensuk et al., 2023; Freitag et al., 2022).

#### 3.2.4 Terahertz time-domain spectroscopy

Terahertz waves refer to electromagnetic waves with frequencies ranging from 0.1 to 10 THz, positioned between the microwave and infrared regions. Terahertz spectroscopy analyzes absorption and refraction during the interaction of terahertz waves with substances. Due to their strong penetration, low ionization energy, and fingerprint characteristics, terahertz waves enable nondestructive pesticide detection. Terahertz time-domain spectroscopy (THz-TDS), a far-infrared band spectroscopy technology based on terahertz radiation, can determine molecular structural information and achieve nondestructive detection of pesticide residues (Cao et al., 2021) and mycotoxins (Hu et al., 2023) by detecting the absorption of molecules at different terahertz frequencies. THz-TDS integrates high penetration and fingerprint recognition for nondestructive detection in multi-layer materials, acquiring multi-dimensional optical parameters in a single scan without complex pretreatment. However, this technology still faces challenges: First, the strong absorption characteristics of water molecules in the terahertz band may easily interfere with the matrix, limiting its applicability to high-humidity medicinal materials (Li Y. et al., 2025); Second, detection sensitivity is constrained by the diffraction limit, leading to insufficient spatial resolution for micrometer-sized contaminants; Finally, the high cost of the equipment restricts its application in on-site rapid detection.

#### 3.2.5 Surface-enhanced Raman spectroscopy

Surface-enhanced Raman spectroscopy (SERS) significantly enhances Raman scattering intensity by adsorbing analytes onto the surfaces of metal colloidal particles or roughened metals (e.g., silver, gold, copper). Currently, two primary theoretical explanations exist for the SERS enhancement effect: electromagnetic field enhancement (EE) and chemical enhancement (CE) (Guo et al., 2024). The predominant factor contributing to EE is surface plasmon resonance (SPR), which redistributes the electromagnetic field intensity around nanoparticles, thereby increasing Raman scattering intensity within the vicinity. CE arises from electronic coupling interactions between the adsorbed molecules and the metal surface, with its main mechanism being charge transfer. Under incident light excitation, orbital electrons in the adsorbed molecules are transferred to unoccupied energy levels of the metal, forming resonance between the molecules and the metal substrate.

SERS exhibits high sensitivity, specific molecular recognition, and real-time, rapid detection capabilities, making it well-suited for on-site applications in fields such as food safety and environmental protection. Zhang et al. (2024b) utilized SERS technology based on tungsten atom-modified gold nanoparticles (STAO-AUNPs) to optimize surface charge and interparticle gaps, thereby enhancing the adsorption of positively charged pesticide molecules and improving detection sensitivity. The detection limits for acetamidine, paraquat, and carbendazim were as low as 0.1 ppb. Zhu et al. (2023) developed a bionic flexible SERS sensor inspired by the mastoid structure of lotus leaves. It enables *in-situ* detection on non-planar surfaces and integrates with portable Raman devices for rapid multi-component analysis of organophosphorus pesticides, such as triazole phosphorus and parathion. Fan et al. (2022) fabricated a Si@Ag@PEI composite functional film substrate for SERS-based detection of SO_2_ residues in ginseng and Salvia miltiorrhiza, with a LOD of 0.25 mg/kg, which is significantly below the pharmacopoeial standard of 10 mg/kg. Wang et al. (2025a) designed and developed a dual-modal aptamer-based sensor utilizing a 3D gold nanoparticle spherical signal array to simultaneously enhance fluorescence and SERS for OTA capture. In combination with magnetic nanoparticles, this approach enabled the specific capture and efficient separation of OTA. The detection limits were determined to be as low as 17.06 fg/mL for SERS and 21.87 fg/mL for fluorescence, respectively. Adade et al. (2024) developed a sensor based on SERS with gold nanoparticles (AuNPs) as the substrate for the detection of benzoyl (b) fluoranthene (BbF) in shrimp (LOD 0.12 ng/mL).

SERS provides novel “fingerprint” information but faces challenges: The uniformity and stability of substrates have a direct impact on signal reproducibility, while the fabrication process of nanostructures is both complex and resource-intensive. Competitive adsorption among multiple components in complex TCM matrices may suppress target substance signals, necessitating improvements in selectivity via techniques such as molecular imprinting or immunoprobes. Quantitative analysis depends heavily on reference material databases and robust algorithms but remains susceptible to background interference, particularly for low-concentration samples (Zhang et al., 2021). Consequently, SERS technology still encounters challenges in achieving accurate quantitative analysis. Optimizing sample pretreatment methods and enhancing substrate selectivity are essential prerequisites for the practical application of SERS in the future.

#### 3.2.6 Hyperspectral imaging

Hyperspectral imaging (HSI) integrates spectroscopy and imaging, acquiring spectral and spatial data to form a 3D data cube. It excels in rapid, precise detection of pollutant residues, enabling the mapping of spatial distribution and chemical composition analysis. Alisaac et al. (2019) used HSI for nondestructive detection of Fusarium infection and mycotoxins in wheat, correlating spectral reflectance with fungal DNA and toxin content (r>0.80). Lu et al. (2024) combined Vis-NIR hyperspectral imaging (400–1734 nm) with multi-task deep learning networks (ENet and HMNet) to enable rapid screening of Scutellaria ecotypes and simultaneous prediction of Cd, Zn, and Pb content, achieving a reduced deviation of 30.61%. Liang et al. (2024) combined HSI technology with an optimized SVM model using spectral preprocessing (WT-TC) and feature extraction (CARS), enabling efficient, nondestructive detection of pesticide residues in tobacco with a classification accuracy of 91.8%. However, HSI has obvious application limitations: massive spectral data require high storage and computing resources, and real-time processing relies on high-performance algorithm optimization; detection sensitivity is limited by spectral resolution and noise level, and its ability to identify trace pollutants is weaker than mass spectrometry technology; the unevenness of the surface and internal structure of TCM can easily lead to spectral interference (Pan et al., 2024).

### 3.3 Inductively coupled plasma mass spectrometry

Inductively coupled plasma mass spectrometry (ICP-MS) utilizes high-temperature plasma to atomize and ionize samples, separates ions by mass-to-charge ratio (m/z), and quantifies them. It offers high sensitivity, rapid analysis, wide dynamic range, and simultaneous detection of major (e.g., Cu, Fe) and ultra-trace elements (e.g., Tl, U), making it ideal for complex TCM matrices. While ICP-MS is an ideal choice for trace element analysis, spectral interferences is a major limitation for multi-element analysis. Polyatomic ions and isobaric overlaps can lead to inaccurate quantification (Lee and Ko, 2023), especially for trace elements in the complex matrix of TCM. Therefore, approaches have been developed to accurate results for a wide range of analyte elements and sample types, including collision/Reaction Cell (CRC) technology and triple quadrupole ICP-MS (ICP-QQQ). Cells filled with inert gasesor reactive gases are placed before the mass analyzer. Collisions or specific chemical reactions selectively remove interfering polyatomic ions while transmitting analyte ions. ICP-QQQ employs two quadrupoles with a CRC in between. The first quadrupole can be set to transmit only the precursor ion of interest, which then reacts in the cell. This provides an unparalleled level of specificity and interference removal, particularly beneficial for ultra-trace analysis in complex TCM extracts. However, high equipment costs and the requirement for professional operation have limited the application of the aforementioned technology (Yang, 2021; Kadar et al., 2011; Li et al., 2023).

### 3.4 Biological detection technology

Biological detection leverages molecular recognition specificity (e.g., antigen-antibody, enzyme-substrate) to precisely identify trace contaminants (pesticides, metals, mycotoxins) in complex TCM matrices, overcoming multi-component interference limitations of physicochemical methods. This approach effectively overcomes the limitations imposed by multi-component interference that are commonly encountered in conventional physicochemical methods. Furthermore, biological detection technology integrates rapid response, on-site applicability, and high-throughput screening capabilities, offering an efficient and reliable solution for the quality control of TCM.

#### 3.4.1 Enzyme inhibition method

The enzyme inhibition method, which relies on the specific inhibitory effect of acetylcholinesterase (AChE) activity, is one of the earliest developed and most widely applied techniques for pesticide residue detection. This method is particularly suitable for organophosphorus and carbamate pesticides (Wang L. F. et al., 2023). By exploiting the specific inhibitory effect on AChE activity, it enables rapid detection via colorimetric reactions or electrochemical signal changes, offering advantages such as simple operation, short detection time, and high cost-effectiveness. Conventional visible-light colorimetry has relatively low sensitivity, which limits the performance of traditional enzyme inhibition methods in detecting low-concentration pesticides. A common strategy to improve sensitivity is replacing spectrophotometry with fluorescence analysis. Fluorescent reporter molecules have distinct excitation and emission spectra that reduce non-specific interference and background noise. As a result, fluorescence-based methods offer significantly higher sensitivity up to one to three orders of magnitude lower detection limits—compared to conventional colorimetric and spectrophotometric approaches. Wang et al. (2019) developed a high-sensitivity fluorescence biosensor for the detection of organophosphorus pesticides (OPs) based on the AChE-regulated “off-on-off” fluorescence strategy (dichlorvos LOD 0.05 ng/mL).

The primary limitations of the enzyme inhibition method include the following aspects: Firstly, the detectable pesticide types are restricted, currently being predominantly applicable to organophosphorus and carbamate pesticides, while exhibiting low sensitivity toward other pesticide classes; Secondly, cholinesterase exhibits poor stability and is prone to inactivation, necessitating low-temperature storage conditions; Thirdly, it is susceptible to interference from endogenous sulfides present in TCM, leading to reduced detection accuracy. To enhance specificity, it is essential to combine appropriate pretreatment methods (Wang W. et al., 2023).

#### 3.4.2 Immunoassay

The immunoassay method is based on the specific binding of antibodies to antigens (or antigens to antibodies) and leverages the high specificity of antibodies to achieve qualitative or quantitative determination of the analyte content through antigen-antibody interactions.

##### 3.4.2.1 Enzyme-linked immunosorbent assay

Enzyme-linked immunosorbent assay (ELISA) quantifies trace contaminants via antigen-antibody binding and enzyme-labeled signal amplification, suitable for rapid pesticide and mycotoxin monitoring. Based on differing reaction principles, ELISA can be categorized into non-competitive and competitive methods. For small molecule compounds such as pesticides and mycotoxins, the competitive detection mode is typically employed, where the detection signal exhibits a negative correlation with the target analyte concentration (Pérez-Fernández et al., 2020).

ELISA has two primary limitations in residue detection: (1) Detection results are susceptible to matrix effects, cross-reactions, and non-specific binding, which may compromise sensitivity. As the key recognition element in ELISA, antibodies play a critical role in enhancing the specificity, affinity, and sensitivity of the assay. Li et al. (2016) developed a high-sensitivity biosensor for 3-5 ring PAHs based on a monoclonal antibody (2G8), achieving a detection limit as low as 0.2 μg/L. Zhao et al. (2019) designed a novel antigen for imidacloprid by exposing the furan group of imidacloprid molecules, thereby improving antibody specificity and sensitivity and resolving the cross-reaction issue encountered in traditional ELISA methods for imidacloprid detection. Wu et al. (2022b) developed a high-affinity monoclonal antibody (^13^C_8_) for carbofuran and its analogues (IC50 0.18 ng/mL). (2) Traditional ELISA primarily employs natural enzymes such as horseradish peroxidase, alkaline phosphatase (ALP), and glucose oxidase (GOx) as signal probes. However, the limited number of natural enzymes that can be directly loaded onto antibodies or antigens restricts the range of detectable enzymes and results in relatively lower output signal intensity, thereby limiting sensitivity. Lyu et al. (2023) developed a two-dimensional Fe-N-C single-atom catalyst (2D Fe-SASC) with peroxidase-like activity and applied it to sensitive immunoassays, achieving high-sensitivity detection of 2,4-dichlorophenoxyacetic acid (2,4-D) with a detection limit of 0.72 ng/mL, offering potential to replace traditional natural enzymes. Tong et al. (2022) developed a novel ELISA method based on M13 phage for OTA detection. The phage serves as both an enzyme carrier and competitive antigen, enabling signal amplification through epitope mimicry and biotin enrichment. AuNP aggregation produces colorimetric and photothermal signals that enhance sensitivity.

##### 3.4.2.2 Colloidal gold immunoassay

Colloidal gold-based immunoassay technology employs colloidal gold as a tracer marker and integrates it with ligands for competitive immune reactions. In a weakly alkaline environment, colloidal gold carries a negative charge and forms strong bonds with the positively charged groups of protein molecules. The aggregation at the binding site enables qualitative or semi-quantitative rapid detection of the analyte. Taking OTA as an example, OTA-BSA conjugates and gold-labeled antibodies are co-immobilized on nitrocellulose membranes. OTA in the sample competes with the immobilized antigen for binding to the gold-labeled antibody. Unbound antibodies form a red band at the test line (T line), with the color intensity inversely proportional to the OTA concentration. Semi-quantitative analysis can be completed within 15 min. Deng et al. (2022) developed colloidal gold immunochromatographic strips using coated antigens and anti-isopropenyl ketone monoclonal antibodies for rapid detection of isopropenyl ketone residues in tobacco, with a visual detection limit of 0.09 mg/kg.

##### 3.4.2.3 Fluorescence immunoassay

Fluorescence immunoassay (FIA) employs fluorescent substances to label antibodies or antigens and quantitatively analyzes target substances by detecting fluorescence signal intensity. After the fluorescently labeled antibody binds to the target toxin, it can either engage in competitive binding with immobilized antigen (competitive method) or form a sandwich complex with coated antibody (sandwich method). Signal intensity is measured using a fluorescence spectrometer or microplate reader. This technology exhibits advantages such as high sensitivity, wide linear range, and suitability for simultaneous detection of trace and high-concentration pollutants. Zhang et al. (2022) developed a time-resolved fluorescence immunoassay for simultaneous detection of zearalenone (ZEN) and deoxynivalenol (DON) in corn and wheat, with detection limits of 7.92 μg/kg for ZEN and 71.90 μg/kg for DON. Wang et al. (2020) established a fluorescence immunoassay based on the fusion of nanobody and alkaline phosphatase for AFB1 detection. Using the Nb-AP fusion protein and phosphate-triggered fluorescence system, the method significantly improved detection sensitivity, achieving a detection limit of 0.12 ng/mL. Jin et al. (2024) utilized high-loading silica to assemble multicolor quantum dots and developed a fluorescence immunoassay for simultaneous detection of OTA, AFB1, FB1, and ZEN in corn, rice, and oats. The method showed excellent linearity in the range of 0.01–100 μg/L, with LODs of 0.0001–0.001 μg/L.

However, FIA depends on precision instruments such as fluorescence spectrophotometers, leading to high detection costs. Additionally, this method is relatively sensitive to environmental factors and susceptible to interference from spontaneous fluorescence in complex herbal matrices, as well as temperature, solvent effects, and scattering light, thereby imposing certain limitations in practical applications.

##### 3.4.2.4 Immunochromatography

Immunochromatography (ICA) method integrates immunological techniques with chromatographic technology, achieving separation and signal conversion of the target substance through lateral chromatographic migration. After the labeled antibody binds to the target substance, it migrates to the detection line (T line), forming a sandwich complex with the immobilized antigen and producing a visible color response. Unbound labeled substances continue migrating to the control line (C line) to verify the system’s effectiveness. The primary advantage of ICA is its rapid detection speed (5–15 min), simple operation, and minimal reliance on complex instruments, making it suitable for on-site screening of external contaminants such as heavy metals, mycotoxins, and pesticide residues. Recent advancements have significantly improved detection sensitivity by incorporating technologies such as quantum dot (QD) labeling. Zhou et al. (2021) developed a QDs-ICA labeling method for rapid and sensitive OTA detection in corn, achieving an LOD of 0.07 ng/mL. The method demonstrated high accuracy and precision, offering a new approach for on-site OTA screening.

However, traditional ICA still faces challenges, including the risk of false positives due to cross-reactions and limited capability for simultaneous multi-component contaminant detection. Zhang et al. (2023c) developed a broad-spectrum monoclonal antibody using a multi-immunogen strategy, enabling rapid detection of eight neonicotinoid insecticides via molecular docking and gold nanoparticle (GNP)-based immunochromatography. The detection limits were 0.76–30.19 μg/kg (ginseng) and 0.87–31.57 μg/kg (tomato), with recovery rates ranging from 72.04% to 120.74%. Recent advances have combined the simplicity and portability of traditional test strips with the high sensitivity and efficient fluorescence intensity recognition capabilities of detection equipment, presenting significant potential for on-site multi-channel rapid detection of small molecule contaminants. Xu et al. (2023) proposed a portable multi-channel detector based on time-resolved fluorescence immunochromatography test strips (TRFIS) for on-site detection of cyprodinil in vegetables. The device can simultaneously scan six TRFIS channels within 30 s and shows high accuracy, with an average coefficient of variation of 2.5% per channel.

#### 3.4.3 Molecular imprinting technology

Molecular imprinting technology (MIT) is an emerging molecular recognition technology, using the target molecule as a template and reacting it with functional monomers under specific conditions to form molecular imprinting polymers (MIPs) with a specific three-dimensional structure and binding sites. After removal of the template molecule, the polymer retains cavities matching the molecule’s shape, size, and functional groups, enabling selective recognition and binding of the target, thereby achieving selective separation and detection of the analyte. MIT offers advantages such as high selectivity, strong stability, low cost, and high sensitivity. Its precise recognition ability allows for accurate differentiation of target pollutants, making it suitable for complex herbal matrix samples. MIPs are chemically stable, maintain performance across pH levels and in organic solvents, and can be reused, reducing detection costs. Mehmandoust et al. (2023) developed a molecularly imprinted polymers/metal-organic framework/gold stabilized on graphite carbon nitride (MIP-Au@MOF-235@g-C_3_N_4_) for fenamiphos detection in vegetables. The limit of detection (LOD) was 7.13 nM, with recovery rates ranging from 94.7% to 107.9%. Chen et al. (2017) synthesized MIPs for organophosphates as SPE adsorbents coupled with GC, achieving recoveries of 87.48%–97.85% in lettuce. Moya-Cavas et al. (2023) optimized the solid-phase extraction conditions of mixed MIPs for zearalenone and fusarium mycotoxins using a stoichiometric method, achieving efficient detection of these toxins in corn, sunflower seeds, and olive oil samples. The detection limits were 5 μg/kg and 2 μg/kg. Castro-Grijalba et al. (2020) combined MIP films with AuNPs for SERS detection of PAHs. The detection limit reached the nM level, which is 100 times more sensitive than non-imprinted plasmonic sensors.

Despite advantages in selectivity and stability, MIT imposes substantial limitations on contaminant detection in TCM. The synthesis process is complex, and achieving reproducibility remains challenging—issues exacerbated by the limited range of suitable functional monomers and cross-linkers available. A critical constraint lies in incomplete template molecule removal during synthesis, which frequently induces template leaching in subsequent analyses, thereby precipitating false positives and compromising quantification accuracy (Brazys et al., 2025). Traditional MIPs, typically synthesized in organic solvents, demonstrate suboptimal performance in aqueous matrices such as TCM extracts, stemming from dominant hydrophobic interactions and competition by water molecules for binding sites (Testa et al., 2022). Furthermore, inherent heterogeneity of binding sites, a direct outcome of conventional bulk polymerization methods, yields low recognition efficiency and binding capacity.

#### 3.4.4 Nucleic acid aptamers

Aptamers are short single-stranded DNA or RNA sequences selected through *in vitro* screening, capable of specifically recognizing target molecules. Using Systematic Evolution of Ligands by Exponential Enrichment (SELEX), aptamers that bind to target pollutants are identified and immobilized on sensor surfaces. When the pollutant binds to the aptamer, it triggers changes in the sensor’s physical or chemical properties, generating detectable signals. Wan et al. (2024) utilized Capture-SELEX to screen high-affinity aptamers for p-hydroxybenzylhydroxysulfate (p-HS) and developed a colorimetric sensor based on these aptamers and gold nanoparticles. The method achieved a detection limit of 1 μg/mL, with recovery rates of 88.5%–105% in potato samples. Yang and Tsai (2022) screened an AFB1-specific aptamer, with a dissociation constant (Kd) of 2.5 µM and no cross-reactivity with other toxins. The aptamer-based sensor demonstrated a wide detection range (50 ppt–50 ppb) and achieved an accuracy rate of 90% in peanut extract samples, showing potential for on-site detection.

Aptamers offer advantages over traditional antibodies, including high-temperature resistance, reversible reconstitution, and flexible chemical modification. However, challenges remain, such as susceptibility to matrix interference and unclear mechanisms of target binding kinetics. Additionally, the SELEX screening process is time-consuming and expensive. Currently, its primary applications are limited to precise laboratory detection, while the rapid screening at TCM production sites requires the development of portable detection devices. In the future, integrating aptamer technology with microfluidic chips and nano-signal amplification techniques could enhance its practicality for simultaneous multi-component and multi-pollutant detection in TCM (Verdian, 2018).

### 3.5 Biosensors

Biosensors integrate biological recognition elements (e.g., enzymes, antibodies, nucleic acid aptamers) with transducers, converting analyte concentration into measurable electrical, optical, or mass-change signals. The core of biosensors comprises two essential components: the biological receptor responsible for the specific recognition of the target analyte and the detection platform that translates the biological response into quantifiable signals. Leveraging their high sensitivity and miniaturization advantages, biosensors enable *in-situ*, real-time monitoring within complex TCM matrices. Recent advances integrate classical principles (enzyme inhibition, immune recognition) with nanomaterials and microfluidics for “recognition-enrichment-amplification,” overcoming sensitivity and throughput limitations. Furthermore, miniaturization facilitates transition from lab to field, offering innovative solutions for the precise monitoring of trace exogenous pollutants in the complex matrix of TCM.

#### 3.5.1 Nanomaterial-based biosensors

Nanomaterials exhibit advantages such as high specific surface area, unique physical and chemical properties, and excellent biocompatibility, which can significantly enhance the performance of sensors. Recent advancements have significantly leveraged the unique optical and electronic properties of metal nanoparticles (MNPs) and quantum dots (QDs) to enable highly sensitive and often direct detection strategies for exogenous contaminants within complex TCM matrices, representing a critical frontier in rapid quality control. The core innovation lies in the direct transduction of the binding event into an easily measurable optical signal (color change or fluorescence change) facilitated by the intrinsic nanoscale properties of these materials, bypassing the need for complex labeling or separation steps often required in conventional assays. Gold and silver nanoparticles (AuNPs, AgNPs) exploit their intense localized surface plasmon resonance (LSPR), which is exquisitely sensitive to changes in the local dielectric environment or nanoparticle aggregation state induced by the specific binding of target contaminants. Zhao et al. (2021b) developed a fluorescence aptamer sensor based on upconversion nanoparticles (UCNPs) and MIL-101(Cr) for the detection of T-2 toxin, achieving a detection limit as low as 0.087 ng/mL, demonstrating a good linear relationship and high recovery rate. QDs can be directly functionalized with biorecognition elements (e.g., antibodies, aptamers) targeting specific contaminants like mycotoxins or residual pesticides. Upon binding the target, direct modulation of the QD fluorescence—through mechanisms like fluorescence resonance energy transfer (FRET), electron transfer, or aggregation-induced quenching—provides a highly sensitive, quantitative signal. Liu et al. (2021) developed a fluorescence “off-on-off” sensor based on BCNO QDs-MnO_2_ nanosheets for the detection of OPs, achieving a detection limit as low as 0.03 ng/mL, with smartphone-readable test strips. Li et al. (2025a) combined hydrophobic deep eutectic solvent (HDES) and carbon quantum dots (CQDs) to build a high-throughput point-of-care serum iron detection system (HT-POCT) based on a machine learning-assisted fluorescence detection platform. By concentrating iron ions and reducing the interference of hydrophilic substances, this system achieved specific recognition of Fe^3+^, with a detection limit as low as 33 nM. Liu et al. (2025) integrates deep eutectic solvents (DES) with MIP to fabricate nanoscale Poly (DES)-MIT sorbents for atrazine removal achieving highly selective recognition and near-quantitative recovery (99.93%).

As a novel class of inorganic materials, nanomaterials possess both the physical and chemical properties characteristic of nanomaterials and catalytic functions similar to enzymes, combining high catalytic efficiency with strong stability and facilitating large-scale production. Chen et al. (2021) developed a competitive bio-barcode immunoassay based on dual-metal Au@Pt nanomaterials for OPs in agricultural products. The Au@Pt nanomaterials exhibit enzyme-like catalytic activity, converting non-fluorescent substrates into fluorescent products without traditional enzyme labeling, thereby significantly lowering the detection limit to 1.47 ng/kg. Lv et al. (2023) developed a colorimetric biosensor based on aptamer amplification for OTA detection using manganese oxide nanoflowers with oxidase-mimicking activity. By enhancing the class-oxidase activity of MnO_2_ nanoflowers through aptamer adsorption, the affinity for chromogenic substrates was improved, achieving highly sensitive OTA detection with a detection limit of 0.069 ng/mL. Tian et al. (2024) discovered that sulfonylurea pesticides enhance the activity of copper-based nanomaterials and combined this with array technology to construct a six-channel sensing array method for selective identification of sulfonylurea pesticides and total concentration detection (with a detection limit of 0.03 μg/mL). Yang et al. (2025b) developed N,O-coordinated single-atom iron-based nanomaterials via a template pre-modification strategy to enhance catalytic efficiency for colorimetric detection of OPs. The method, combined with a portable smartphone sensor, achieved a detection limit of 0.85 ng/mL.

#### 3.5.2 Microfluidic-based biosensors

Microfluidics enables the precise manipulation of minute fluid volumes (ranging from nanoliters to picoliters) through microscale channel networks. By integrating sample pretreatment, reaction, separation, and detection modules onto a single chip, it facilitates the miniaturization and automation of the detection process. This miniaturized design not only substantially reduces the detection cycle but also increases detection throughput via multi-channel fluid control, thereby enabling the transfer of laboratory-level precision to on-site rapid testing scenarios. Hefner et al. (2024) developed a novel microfluidic paper-based analytical device (mPAD) for on-site detection of Ziram, with a detection limit of 1.5 ppm, which enables rapid transport of pesticide samples through capillary channels, effectively avoiding the adsorption of pesticides in microchannels. Wu et al. (2025) developed an electrochemical aptamer sensor based on a comb-shaped microfluidic chip for the simultaneous detection of AFB1 and DON, featuring enhanced binding efficiency. This sensor achieves detection limits of 7.74 pg/mL for AFB1 and 0.172 ng/mL for DON, demonstrating excellent specificity, repeatability, and stability. It provides an efficient new tool for simultaneous on-site detection of multiple toxins.

#### 3.5.3 Dual-mode biosensors

Dual-mode biosensor integrates two independent detection mechanisms (e.g., optical-electrochemical, colorimetric-fluorescent) to achieve synchronous output and mutual validation of multiple signals, representing a significant technological advancement in the detection of exogenous pollutants in TCM. Its key advantage lies in enhancing detection reliability through signal complementarity: first, data from the dual channels can be cross-validated, effectively mitigating risks of false positives/negatives caused by matrix interference or environmental fluctuations; second, differences in sensitivity between detection modes broaden the linear detection range, with colorimetric methods suitable for rapid screening and fluorescent/electrochemical methods enabling trace quantification. Compared to single-mode sensors, the dual-mode design markedly improves the resolution of exogenous pollutants in complex TCM matrices. Wang et al. (2024b) developed a colorimetric-fluorescent dual-mode immunosensor based on spot-silver-doped nano-hybrids for ultra-sensitive detection of OTA. The sensor achieves colorimetric rapid screening (LOD 23.5 pg/mL) and fluorescence-based high-sensitivity quantification (LOD 0.99 pg/mL), with recovery rates ranging from 86% to 118% in complex matrices such as corn and flour, demonstrating significantly improved interference resistance and reliability. Zha et al. (2021) designed a dual-mode immunosensor based on biotin-labeled IgG-modified gold nanoparticles for detecting chloroacetanilide herbicides, achieving dual signal outputs of colorimetry and fluorescence. This sensor enables simultaneous detection via smartphone and fluorescence spectrometer, significantly enhancing both detection sensitivity and convenience.

## 4 Bottlenecks and challenges

### 4.1 Balance between risk management and inspection expenses

The cornerstone of safety surveillance for exogenous contaminants lies in precision risk-based control, which is fundamentally dependent on robust regulatory frameworks and validated analytical capabilities. Escalating stringency in pharmacopeial limits and technical guidelines for pesticide residues, heavy metals, and mycotoxins—as outlined in Chinese Pharmacopoeia General Chapters 0212/2341 and aligned with ICH Q3D—demands integrated analytical performance encompassing multi-analyte coverage, ultra-trace sensitivity, and metrological-grade accuracy. These capabilities are essential for generating comprehensive and reliable surveillance data to support evidence-based risk assessment. However, operational realities necessitate equilibrium between regulatory compliance and cost efficiency. High-complexity laboratory infrastructures (e.g., LC-MS/MS) entail significant operational expenditures, requiring advanced instrumentation, specialized technical expertise, and controlled environments, collectively posing critical scalability challenges.

### 4.2 Complexity of the herbal matrix

The core challenge in the detection technology for exogenous contaminants in TCM stems from the inherent complexity of the herbal matrix. Natural compounds such as polyphenols, polysaccharides, pigments, and structural analogues present in TCM form a mixed system with highly overlapping physicochemical properties and contaminants, hindering efficient purification via traditional methods and causing co-elution in chromatography. Components like alkaloids, tannins, and pigments induce matrix effects in MS ionization, leading to signal suppression or enhancement of target contaminants and introducing severe matrix interference and false positives during detection (Wu and Ding, 2023). Additionally, for structurally similar pollutants (e.g., carbofuran and its metabolite 3-hydroxycarbofuran), conventional mass spectrometry multiple reaction monitoring (MRM) is susceptible to chromatographic co-elution and qualitative deviations due to the high similarity of fragment ions. High-resolution mass spectrometry can distinguish these compounds through precise mass numbers and isotope distribution comparisons but requires stringent optimization of instrument parameters.

### 4.3 Synergistic challenges of sensitivity and accuracy in trace analysis

The detection of trace exogenous contaminants in TCM must simultaneously achieve ultra-low detection limits and high-precision quantitative requirements, posing significant challenges to technical performance. Enhancing detection sensitivity often comes at the cost of narrowing the quantitative linear range or increasing the risk of false negatives. Consequently, achieving a balanced optimization between high sensitivity and high accuracy has emerged as a critical scientific issue. High-sensitivity methods, such as SERS and biosensors, are susceptible to interference from endogenous pigments and alkaloids in the herbal matrix, potentially leading to target signal suppression or reduced specificity, with an associated risk of false negatives. The use of nanomaterials can significantly lower the detection limit; however, their narrow linear range (10^–8^ to 10^–6^ M) (Wen et al., 2025) and suboptimal repeatability indicators make it challenging to meet pharmacopoeia standards.

### 4.4 Demand for rapid and portable on-site detection

Rapid detection technologies (e.g., LFIA) encounter significant application bottlenecks. The antigen-antibody reaction and chromatographic process are highly sensitive to fluctuations in environmental temperature and humidity (e.g., impacting antibody activity and membrane capillary flow rates) as well as matrix interference, leading to poor signal stability and reproducibility. Furthermore, the visual colorimetric judgment mode is prone to subjective errors and environmental light interference, making it challenging to achieve accurate quantification of trace contaminants. Consequently, its quantitative detection limit is approximately 1–2 orders of magnitude higher than that of laboratory-based methods. These limitations require confirmation of positive results through standard laboratory techniques, which restricts the technology’s utility in making immediate on-site decisions.

### 4.5 Transformation bottleneck of full-process digital detection

While digital technologies offer significant potential for transforming the detection of exogenous contaminants in TCM, encompassing intelligent data collection (portable spectrometers), AI-driven analysis (such as machine learning for chromatography/mass spectrometry, smartphone image recognition for test strips), the digital transformation of the entire detection process still encounters critical challenges. The foremost challenges hindering this digital transformation include: 1) the lack of standardized data formats and protocols, which severely impedes the integration and interoperability of multi-source information across the detection workflow. 2) insufficient generalization capabilities of existing AI models when confronted with the extreme complexity and variability inherent in TCM matrices, leading to unreliable analytical results and limiting model deployment. These fundamental issues pose significant barriers to realizing a truly effective and reliable digitalized detection process.

## 5 Future perspectives

Overall, the monitoring of exogenous contaminants in TCM based on risk control should be advanced through two complementary approaches: the continuous refinement of regulatory methods and the development of rapid on-site detection technologies. First, in accordance with domestic and international pharmacopoeia standards and technical guidelines, the coverage of detection indicators should be expanded, and multi-residue and high-throughput detection technologies further optimized to enhance the applicability and authority of standard methods. Second, by integrating historical laboratory detection data, rapid and user-friendly on-site screening tools (test strips and sensors) should be developed to target specific contaminants present in high-risk varieties. Following the risk-stratified management approach, a comprehensive quality control system encompassing both “standard methods” and “on-site rapid screening” should be established. This will enable precise monitoring of exogenous contamination in TCM while ensuring risks remain within acceptable limits.

Building upon this foundational risk-based monitoring framework, overcoming the inherent technical challenges posed by complex matrices is paramount. Owing to the complex matrix characteristics of TCM, there exists an inherent trade-off between the sensitivity and accuracy of trace detection methods, which imposes higher demands on the development of these methods. Consequently, the future advancement in rapid detection technologies should prioritize the following directions: First, enhancing the specificity of pretreatment techniques by designing materials based on highly specific recognition elements, such as molecular imprinting solid-phase microextraction (Thati et al., 2024; Yang S. et al., 2025), to reduce matrix interference through targeted adsorption; employing novel chromatographic stationary phases (Zeng et al., 2022) to improve the separation efficiency of trace contaminants in complex systems. Second, developing anti-interference high-sensitivity detectors, such as utilizing nanomaterials (such as AgNPs) to amplify the signal, thereby significantly improving both specificity and sensitivity. Third, integrating chemometrics with AI algorithms to calibrate systematic errors and ensure the reliability of quantitative results. Peng et al. (2025) combined smartphone imaging with AI algorithms (such as YOLOv8) enables on-site high-throughput analysis. The successful development of these advanced core detection technologies will be crucial for enabling the next-generation of practical rapid on-site tools.

To fully leverage the potential of these technological advancements and address the bottlenecks of digital transformation, the integration of hardware and software into intelligent platforms is essential. An AI-driven automated detection platform should be implemented to enable end-to-end intelligent management from sample preparation through data analysis to result interpretation. Hardware development requires high-precision Robot Operating System (ROS)-based automation modules for sample weighing and extraction pretreatment. Concurrently, software solutions must incorporate multimodal data fusion algorithms integrating spectroscopic, mass spectrometric, and imaging information to enhance identification accuracy in complex matrices. For example, a one-dimensional convolutional neural networks (CNN) architecture driven by transfer learning has been utilized to increase the accuracy of SERS pesticide classification to 99.75%, providing a paradigm for AI to dynamically calibrate spectral errors in complex matrices (Zhu et al., 2021). Future implementations could leverage such AI algorithms to complete background subtraction, automatic interpretation, and precise quantitative analysis, thereby effectively eliminating subjective errors. However, mitigating overfitting risks arising from insufficient feature representation in pre-training data remains crucial (Hu et al., 2022).

Beyond detection, the future requires a paradigm shift toward active intervention and integrated knowledge management. Emerging dual-functional platforms that synchronize contaminant identification and on-site degradation are critical to transcend the traditional “test-and-remove” passive paradigm. For instance, metal-organic framework (MOF) nanosystems enable stimuli-responsive pesticide detection and controllable photocatalytic degradation, ideal for on-site remediation of pesticide-contaminated TCM (Liu et al., 2023). Besides, semiconductor-metal hybrid substrates achieve ultrasensitive SERS detection coupled with >95% photodegradation efficiency under simulated sunlight, while maintaining recyclability over five cycles in complex herbal samples (Wen et al., 2025). Future research should prioritize adapting these platforms to TCM matrices while ensuring scalability and regulatory compliance.

Complementing these technical innovations and enabling truly intelligent risk control, establishing a specialized TCM contaminant knowledge ecosystem is the ultimate goal. This infrastructure should incorporate databases with pollutant toxicity profiles, analytical methodologies, and regulatory limits, utilizing natural language processing for continuous knowledge integration. Machine learning algorithms can subsequently predict accumulation patterns and generate risk assessment models, while blockchain-enabled platforms provide immutable data traceability, geospatial risk mapping of medicinal material origins, and regulatory decision support through integrated detection-assessment-warning functionalities. This holistic ecosystem represents the future of proactive, data-driven quality assurance for TCM.

## 6 Conclusion

This review establishes a comprehensive technical pathway for exogenous contaminant management in TCM, spanning risk identification to preventive control. We systematically characterize major contaminant sources and hazard profiles and critically evaluate core detection technologies. Key challenges persist, however, including matrix complexity, inherent sensitivity-accuracy trade-offs in trace analysis, limitations in field-deployable technologies, and delays in digital transformation. To overcome these barriers, an integrated framework leveraging advanced detection platforms, intelligent data analytics, and interdisciplinary methodologies is essential. Such integration will transform TCM quality control from passive response to proactive prevention. Successful implementation promises enhanced safety assurance for clinical use, accelerated alignment with international standards to overcome technical barriers, and the establishment of intelligent, portable quality control systems. Ultimately, this synthesis provides robust scientific support for strengthening global regulatory oversight of TCM supply chains.
